# Spatial Measures of Urban Systems: from Entropy to Fractal Dimension

**DOI:** 10.3390/e20120991

**Published:** 2018-12-19

**Authors:** Yanguang Chen, Linshan Huang

**Affiliations:** Department of Geography, College of Urban and Environmental Sciences, Peking University, Beijing 100871, China

**Keywords:** entropy, fractal, scaling, scale dependence, spatial heterogeneity, urban land use

## Abstract

One type of fractal dimension definition is based on the generalized entropy function. Both entropy and fractal dimensions can be employed to characterize complex spatial systems such as cities and regions. Despite the inherent connection between entropy and fractal dimensions, they have different application scopes and directions in urban studies. This paper focuses on exploring how to convert entropy measurements into fractal dimensions for the spatial analysis of scale-free urban phenomena using the ideas from scaling. Urban systems proved to be random prefractal and multifractal systems. The spatial entropy of fractal cities bears two properties. One is the scale dependence: the entropy values of urban systems always depend on the linear scales of spatial measurement. The other is entropy conservation: different fractal parts bear the same entropy value. Thus, entropy cannot reflect the simple rules of urban processes and the spatial heterogeneity of urban patterns. If we convert the generalized entropies into multifractal spectrums, the problems of scale dependence and entropy homogeneity can be solved to a degree for urban spatial analysis. Especially, the geographical analyses of urban evolution can be simplified. This study may be helpful for students in describing and explaining the spatial complexity of urban evolution.

## 1. Introduction

Urban systems indicate both cities as systems and the systems of cities. A city as a system is the concept of an individual city, and belongs to intraurban geography; a system of cities is a concept of an urban network, and belongs to interurban geography [1]. Both cities and systems of cities proved to be self-organizing complex spatial systems [2,3,4]. Complex systems can be described with entropy [5,6,7], including Hartley’s macrostate entropy, Shannon’s information, and Renyi’s generalized entropy. Unfortunately, in many cases, entropy values depend on the scale of measurement [8]. If we study a city as a system, the entropy values in different years (times) may be incomparable; if we research a system of cities, the entropy values of different cities (elements or subsystems) may be incomparable. Spatial scales of measurements can be reflected by the different definitions of zonal systems, resolution ratios of remote sensing images, linear sizes of grids, and so on. The scale dependence of entropy influences the effect of spatial analysis on urban systems. The conventional mathematical modeling and quantitative analyses relies heavily on characteristic scales. Scale dependence suggests that no valid characteristic scale can be found. One method to solve this problem is to replace entropy with fractal dimension in light of the inherent relationship between entropies and fractal dimensions [8,9,10]. Fractals suggest the scaling invariance behind the scale-dependence of spatial entropy in urban systems.

Fractal dimension is the basic parameter for describing self-similar patterns and processes. A fractal has three typical properties: scaling law, fractional dimension, and entropy conservation law [11]. Scaling law implies the scale invariance of the spatial measurement of fractal systems, and entropy conservation suggests that the spatial heterogeneity cannot be effectively reflected by entropy values. On the other hand, there are two ways to define fractal dimension. One is based on entropy functions, and the other is based on correlation functions. These two ways are equivalent to one another, but the angles of view are different. Based on entropy functions, the models are expressed as logarithmic functions, while when based on the correlation function, the models are expressed as power functions. Where spatial correlation is concerned, fractal systems have no characteristic scales; while where spatial entropy is concerned, the fractal dimension just represents the characteristic value of entropy [12]. This suggests that if entropy values depend on the scale of spatial measurement, we can convert the entropy values into fractal dimension values to avoid the scale dependence. Based on generalized entropy, we can obtain multifractal parameter spectrums, and thus characterize the spatial heterogeneity of cities. This paper is devoted to examining the approaches of converting generalized entropy measurements to multifractal measures for the scale-free spatial analysis of fractal urban phenomena. By doing so, the process of spatial analysis of urban systems is simplified, and its efficiency is improved. The remaining parts are organized as follows. In Section 2, the relationships between entropies and fractal dimensions are illustrated from the views of scale dependence and spatial heterogeneity. In Section 3, an empirical analysis is made by means of the city of Beijing, the national capital of China, to verify the theoretical inferences. In Section 4, several related questions are discussed, and finally, the discussion is concluded by summarizing the main points of this work.

## 2. Theoretical Models

### 2.1. Generalized Entropy and Fractal Dimension

In a regular fractal, the complete parts that are similar to the whole are called fractal units. In the literature, fractal units are also called fractal copies [13]. A fractal system is a hierarchy of infinite levels with a cascading structure [14,15,16,17,18,19,20]. A fractal unit is a fractal subset or fractal subsystem at a given level. A fractal structure bears no characteristic scale, and cannot be described with the conventional measures such as length, area, and volume. In other words, the common measures of a fractal system depend heavily on the scales of measurement. The effective measurement of describing fractals is fractal dimension. To understand fractals, we must clarify the three properties of fractal systems: scaling law, fractal dimension, and entropy conservation. First of all, fractal systems follow the scaling law, which indicates some type of scale invariance. Scaling relations can be expressed as a functional equation as below [14,17]: T*f*(*x*) = *f*(*λx*) = *λ^b^f*(*x*), where *f*(*x*) represents a function of variable *x*, **T** denotes an operator of dilation-contraction transform (scaling transform), *λ* refers to scale factors, and *b* is the scaling exponent. In mathematics, if a transform **T** is applied to a function *f*(*x*), and the result is the function *f*(*x*) multiplied by a constant *C* (e.g., *C* = *λ^b^*), then we will say that the function *f*(*x*) is the eigenfunction under the transform **T**, and the constant *C* is the corresponding eigenvalue. The concept of eigenfunctions is a generalization of eigenvector in linear algebra. This implies that a fractal model is just an eigenfunction of scaling transform, and the fractal dimension is associated with the eigenvalue *λ^b^*. The solution to the functional equation is always a power function. Thus, a fractal is often formulated by a power law.

Next, fractal systems often bear fractal dimensions, which is usually a fractional dimension greater than its topological dimension. In Euclidean geometry, a point has zero dimensions, a line has one dimension, a plane has two dimensions, and a body has three dimensions. However, generally speaking, a fractal object cannot be characterized by the integer dimension. In many cases, the integer dimension is replaced by a fractional dimension that comes between zero and three. The fractal dimension of a geometric object is defined as a dimension that is strictly greater than the topological dimension of the object [14]. The fractal dimension can be defined by the scaling exponent *b*. Let’s see a simple fractal model, *N*(*r*) = *N*_1_*r*^−*D*^, in which *r* is the scale of measurement, e.g., the linear size of boxes, *N*(*r*) is the number of fractal copies based on the scale *r*, e.g., the number of non-empty boxes, *N*_1_ refers to the proportionality coefficient, and *D* refers to the fractal dimension. Based on the box-counting method, the fractal parameter satisfies the following condition: *d*_T_ < *D* < *d*_E_, where *d*_T_ refers to the topological dimension of a fractal object, and *d*_E_ is the Euclidean dimension of the embedding space in which the fractal object exists.

Further, fractal systems follow the law of entropy conservation. This is very important for us to understand fractals. Fractal systems can be described by a transcendental equation as follows [21,22]:

(1) $$\sum_{i=1}^{N(r)}P_{i}{\left(r\right)}^{q}r_{i}^{(1-q)D_{q}}=1$$

where *P_i_* is the growth probability of the *i*th fractal unit, *r_i_* is the linear size of the *i*th fractal unit, *q* denotes the order of moment, and the exponent *D_q_* represents the generalized correlation dimension [18]. For a monofractal, i.e., a simple self-similar fractal, we have, *D_q_* ≡ *D*_0_; for a self-affine fractal, different directions have different fractal dimension values, and for a given direction, we have *D_q_* ≡ *D*_0_. However, a multifractal system is more complex. Different parts of a multifractal system have different characters, and can be described with different fractal dimension values. To simplify the process of spatial measurement, the varied linear scales *r_i_* can be substituted with a unified scale *r*. For example, based on the box-counting method, the unified scale, *r*, can be represented by the linear sizes of boxes. Thus, Equation (5) can be re-written as [17,18]:

(2) $$\sum_{i=1}^{N(r)}P_{i}{\left(r\right)}^{q}=r^{(q-1)D_{q}}=r^{\tau (q)}$$

where:

(3) $$\tau (q)=(q-1)D_{q}$$

is termed the mass exponent of a multifractal structure [17,18]. The generalized correlation dimension and the mass exponent compose the global parameters of multifractal description. Suppose that the linear size of boxes *r* approaches the infinitesimal. Taking the natural logarithms on both sides of Equation (3) yields the generalized dimension [21,22,23,24]:

(4) $$D_{q}=-\frac{M_{q}(r)}{\ln r}=\frac{1}{q-1}\frac{\ln\sum_{i=1}^{N(r)}P_{i}{\left(r\right)}^{q}}{\ln r}$$

in which *M_q_*(*r*) represents the *q*th order Renyi entropy based on scale *r*, that is:

(5) $$M_{q}(r)=-D_{q}\ln r=\frac{1}{1-q}\ln\sum_{i=1}^{N(r)}P_{i}{\left(r\right)}^{q}$$

This suggests that the generalized correlation dimension is just the characteristic value of Renyi entropy based on spatial scales [25]. A one-parameter family of normalized measures *μ*(*q*) in which the probabilities in the boxes of linear size *r* can be constructed as follows [26,27]:

(6) $$\mu_{i}(r)=\frac{P_{i}{\left(r\right)}^{q}}{\sum_{i=1}^{N(r)}P_{i}{\left(r\right)}^{q}}$$

If the linear size *r* of boxes is infinitely small, we will have two local parameters of multifractals [26,27]:

(7) $$\alpha (q)=-\frac{S_{q}(r)}{\ln r}=\frac{\sum_{i=1}^{N(r)}\mu_{i}(r)\ln P_{i}(r)}{\ln r}$$

(8) $$f(\alpha )=-\frac{H_{q}(r)}{\ln r}=\frac{\sum_{i=1}^{N(r)}\mu_{i}(r)\ln\mu_{i}(r)}{\ln r}$$

where *α*(*q*) denotes the *q*th order singularity exponent of the *i*th multifractal subset, *f*(*α*) refers to the corresponding fractal dimension of the fractal subset [21,28], *Hq* is the generalized Shannon entropy, and *Sq* is a mixed entropy, which relates the global level to the local levels of multifractal systems. Concretely speaking, we have:

(9) $$S_{q}(r)=-\sum_{i=1}^{N(r)}\mu_{i}(r)\ln P_{i}(r)$$

(10) $$H_{q}(r)=-\sum_{i=1}^{N(r)}\mu_{i}(r)\ln\mu_{i}(r)$$

The local parameters can be associated with the global parameters by the Legendre transform [21,26,27,28], that is:

(11) $$\alpha (q)=\frac{d\tau (q)}{dq}=D_{q}+(q-1)\frac{dD_{q}}{dq}$$

(12) $$f(\alpha )=q\alpha (q)-\tau (q)=q\alpha (q)-(q-1)D_{q}$$

If *D_q_* is termed a generalized correlation dimension describing the global features of multifractal sets, then *f*(*α*) can be termed the generalized information dimension reflecting the local features of the multifractals. It can be proved that the Renyi entropy, generalized Shannon entropy, and mixed entropy can be connected by the Legendre transform.

### 2.2. Scale Dependence and Entropy Conservation of Fractal Urban Systems

Global multifractal parameters are defined on the basis of the scaling relation between Renyi entropy and the corresponding measurement scales. The parameter values of multifractal systems such as cities based on a given approach (e.g., the box-counting method) depend on the scope of the study area (size, central location) [8]. In fact, the commonness between entropy and fractal dimension lies in that both the entropy values and fractal dimension values depend on the method and study area. The advantage of entropy over fractal dimension is that entropy can be applied to measuring both Euclidean structures and fractal structures, while fractal dimension can only be applied to characterizing fractal structures [25]. Compared with entropies, fractal dimensions have two advantages. One is that fractal dimension values do not depend on the linear scale of measurement [8,15,16]; the other is that fractal dimension values can reflect the local feature of random multifractal systems [10,29]. The basic property of fractals is entropy conservation; that is, for a given level of a fractal hierarchy, different fractal units have the same entropy value. The entropy values of the fractal units at a given level in a fractal system depend on the growth probability distribution, but are independent of spatial scales. This implies that entropy cannot be used to describe the local features of different parts of a multifractal system of cities. In other words, entropies cannot reflect the spatial heterogeneity of a complex system. However, different fractal units have different fractal dimension values, which depend on both the growth probability distribution and spatial scales. In this sense, the fractal dimension represents a feasible measure of spatial heterogeneity.

For random multifractals such as cities, which are in fact prefractals, we cannot identify entire fractal units; thus, both entropy and fractal dimension depend on the size and central location of the study area. As we know, the entropy values of a system rely on two factors: one is the number of elements (*N*), and the other is the uniformity or homogeneity of the elements’ distribution. The size distribution of elements is reflected by the probability structure, i.e., the difference of *P_i_* values. For a homogeneous system (say, a regular monofractal object), if we enlarge the size of the study area, the entropy value will increase, but the location has no significant influence on the result. Meanwhile, for a heterogeneous system (say, a random multifractal object), both the size and location of the study area will impact the entropy values: different area sizes indicate different element numbers (*N*), and different locations imply different probability distribution patterns of elements (*P_i_*).

**Figure 1:**
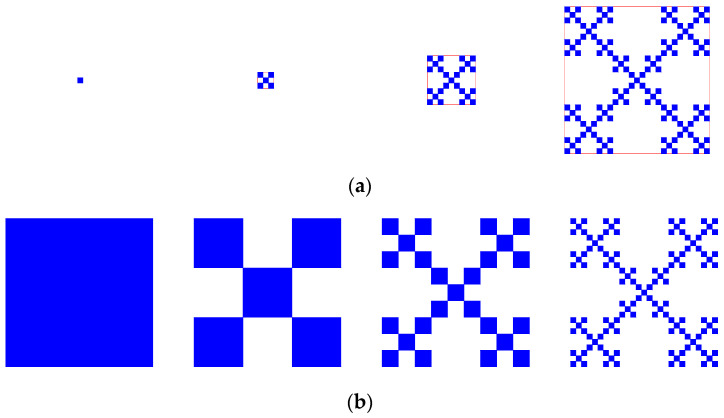
A regular growing monofractal that bears an analogy with urban growth. (**a**) Monofractal growth; (**b**) Monofractal generation. Note: A monofractal possesses only one scaling process, and is also termed “unifractal” in literature. Figure 1a represents the variable scale of measurement based on the variable size of the study area, and Figure 1b represents the variable scale of measurement based on a fixed size of the study area.

It is easy to demonstrate that the entropy values of a monofractal system depend on the size of the study area or scale of measurement. Let’s see two simple examples, which are based on a regular fractal (Figure 1). The fractal was put forward by Jullien and Botet [20] to reflect fractal growth, and became well known due to the work of Vicsek [13]. So, it was termed Vicsek’s fractal, representing an embodiment of Stigler’s law of eponymy [30]. This growing fractal was often employed to act as a simple fractal model of urban growth [15,16,31,32,33]. First, entropy value depends on the size of the study area. Please see the following regular growing fractal (Figure 1a). The first four steps represent a process of a growing prefractal. Different steps reflect the different potential sizes of the study area. The first step is special, and the results are outliers. You can see that the entropy values depend on the study area, but the fractal dimension value is certain. From the first step on, the entropy values and fractal dimensions are listed as below:

- **Step 1**: entropy *H* = 0; fractal dimension *D* = 0. For a point, the fractal dimension value can be obtained by L’Hospital’s rule.
- **Step 2**: entropy *H* = ln(5) =1.6094 nat; fractal dimension *D* = ln(5)/ln(3) = 1.465.
- **Step 3**: entropy *H* = ln(25) = 3.2189 nat; fractal dimension *D* = ln(25)/ln(9) = 1.465.
- **Step 4**: entropy *H* = ln(125) = 4.8283 nat; fractal dimension *D* = ln(125)/ln(27) = 1.465.
- ….

Second, entropy value also depends on the scale of measurement. Now, let’s see the following regular growing fractal (Figure 1b). For this figure, different steps reflect different linear scales of measurement. The first step is special, and the results are outliers, too. The entropy values depend on the linear size, but the fractal dimension value is still certain. The entropy values and fractal dimensions are listed below:

- **Step 1**: entropy *H* = 0; fractal dimension *D* = 2. For a surface, the fractal dimension can be obtained by L’Hospital’s rule.
- **Step 2**: entropy *H* = ln(5) = 1.6094 nat; fractal dimension *D* = −ln(5)/ln(1/3) = 1.465.
- **Step 3**: entropy *H* = ln(25) = 3.2189 nat; fractal dimension *D* = −ln(25)/ln(1/9) = 1.465.
- **Step 4**: entropy *H* = ln(125) = 4.8283 nat; fractal dimension *D* = −ln(125)/ln(1/27) = 1.465.
- ….

For different fractal units in a given level (step), the entropy value and fractal dimension value are both certain; that is, they are constant values (Table 1).

**Figure 2:**
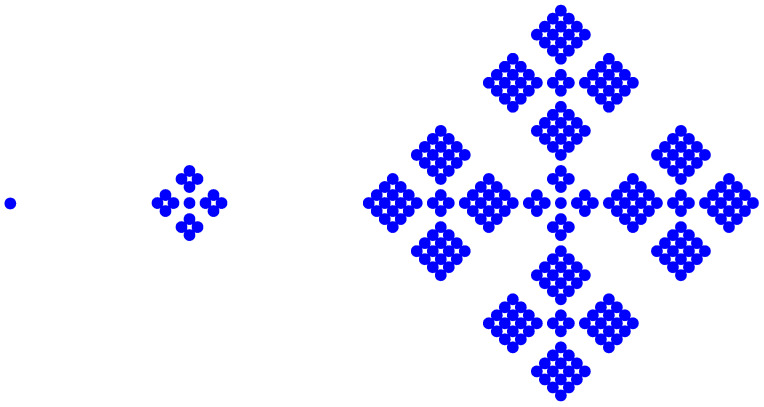
A regular growing multifractal that bears an analogy with urban growth. Note: To illustrate the multifractal, Vicsek [13] proposed this fractal, with two different scales in the generator.

The spatial structure of multifractal systems differs from that of simple fractal systems. For the multifractal systems, entropy values depend on the size and location of the study area, as well as on the scale of measurement. Let’s see an example of the spatial heterogeneity and entropy conservation of multifractals. The following regular growing multifractals are well known for many fractal scientists and some urban geographers (Figure 2). The first step is special, and the results are outliers, too. The entropy value depends on the linear size, but the box fractal dimension value is certain. From the second step onwards, the entropy values and fractal dimensions are listed as below:

- **Step 1**: entropy *H* = 0; fractal dimension *D* = 0.
- **Step 2**: entropy *H* = −ln(1/17)/17 − 4 × 4 × ln(4/17)/17 = 1.5285 nat; box dimension *D* = ln(1/17)/ln(1/5) = 1.7604.
- **Step 3**: entropy *H* = −ln(1/289)/289 − 8 × 4 × ln(4/289)/289 − 16 × 16 × ln(16/289)/289 = 3.0569 nat; box dimension *D* = ln(1/289)/ln(1/25) = 1.7604.

However, for different fractal units, entropy values are constant, but fractal dimension are different. In fact, for a multifractal object, different parts have different local fractal dimensions. The first three steps represent a multi-scaling prefractal. For example, for the second level of the third step, the five parts have two fractal dimension values. For the central part, the box dimension is *D* = ln(17/289)/ln(2/25) = 1.7604; for the other four parts, the box dimension is *D* = ln(68/289)/ln(10/25) = 1.5791. However, different parts have the same entropy values: entropy *H* = −ln(1/17)/17 − 4 × 4 × ln(4/17)/17 = 1.5285 nat (Table 2).

### 2.3. Entropy-Based Fractal Dimension Analysis

According to the above analysis based on regular fractals, we can find two properties of fractal systems. First, the entropy value of a fractal system depends on the scale of measurement, but the fractal dimension is independent of the scales. For both simple fractals and multifractals, different steps represent different measurement scales. For monofractals, based on a certain method, the fractal dimension value is unique. However, for multifractals, different parts have different fractal dimension values. In contrast, for a given part of a multifractal system, the fractal dimension value does not depend on the measurement scales. Second, different fractal units share the same entropy value. The structure of a simple fractal is homogenous, and a fractal unit is the same as the other fractal unit. The entropy value of each fractal unit is the same. On the contrary, the structure of multifractals is heterogeneous, and one fractal unit may be different from another fractal unit. Despite the differences between fractal units, the entropy value of each fractal unit is still the same. However, different fractal units may have different fractal dimension values. This indicates that the fractal dimension of each part does not depend on measurement scales, but rather relies on local structure. Therefore, we can substitute fractal dimension for entropy to make a spatial analysis of cities if one of the following two cases appears. One is that the measurement results depend on scales, and the other is that spatial heterogeneity must be taken into consideration.

In urban studies, it is convenient to transform spatial entropy into multifractal spectrums. The process is as follows. (1) Transform Renyi entropy *M_q_* into global correlation dimension *D_q_* and mass exponent *τ*(*q*). It is easy to define global multifractal dimensions based on Renyi entropy, which are applied to global spatial analyses. The global parameters comprise the generalized correlation dimension and mass exponent. See Equations (1)–(5). (2) Convert the global parameters into local multifractal parameters by Legendre transform. The local parameters, including the local fractal dimension *f*(*α*) and the corresponding singularity exponent *α*(*q*), can be used to make partial spatial analysis. See Equations (6)–(10). (3) Substitute the spatial analysis by moment order analysis. In practice, it is difficult to distinguish the different spatial units of a random multifractal object from one another. A clever solution is to use moment analysis to replace local analysis. Mapping the parameter information of different spatial units into different orders of moment, *q*, we will have multifractal parameter spectrums. A multifractal spectrum based on moment orders can be treated as the result of local scanning and sorting for a complex system [11,34].

## 3. Empirical Analysis

### 3.1. Study Area and Methods

**Figure 3:**
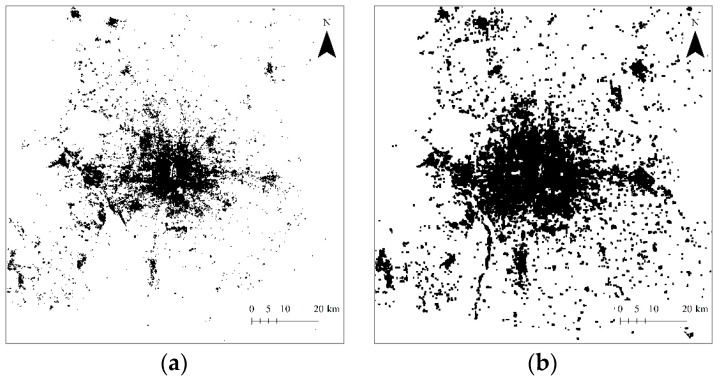

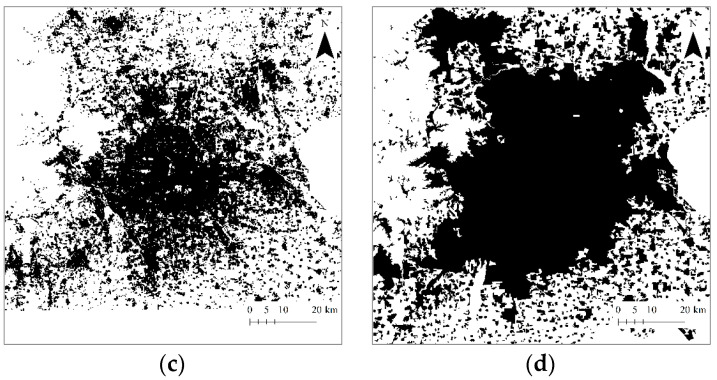
Four typical images of Beijing’s urban land-use patterns. (**a**) 1984; (**b**) 1994; (**c**) 2006; (**d**) 2015.

In this section, we will apply entropy measures and fractal dimension to urban form and growth. Urban form can be reflected and represented by urban population distributions, urban land-use patterns, urban transport networks, and so on [15]. The study area of this work is the urban agglomerations of Beijing city, the national capital of China, and the researched object is urban land use. The datasets came from the remote sensing images of four years, that is, 1984, 1994, 2006, and 2015 (Figure 3). A number of thematic mapper (TM) images of Landsat with a ground resolution of 30 meters of Beijing from the National Aeronautics and Space Administration (NASA) (1984, 1994, 2006) and Institute of Remote Sensing and Digital Earth (ISDE) of the Chinese Academy of Sciences (CAS) (2015) are available for spatial analysis [29,34]. The functional box-counting method can be employed to measure the Renyi entropy and calculate multifractal parameters. This method was originally proposed by Lovejoy et al. [35] to estimate the fractal dimension of radar rain distribution. Later, Chen [36] improved the method and used it to measure the fractal dimension of urban systems. The original functional box-counting method is based on the largest box with an arbitrary area [35], while the improved functional box-counting method is based on the largest box with the measure area of an urban envelope [36]. This improved method is also termed the Rectangle Space Subdivision (RSS) method [29,37]. Where studies on fractal cities are concerned, the improved functional box-counting method bears firm theoretical basis. On the one hand, its geometrical basis of RSS is the recursive subdivision of space and the cascade structure of hierarchies [15,38]; on the other, its mathematical basis is the transformation relation between the power laws based on dilation symmetry and the exponential laws based on translational symmetry [39].

The procedure of data extraction and parameter estimation comprises four steps.

**Figure 4:**
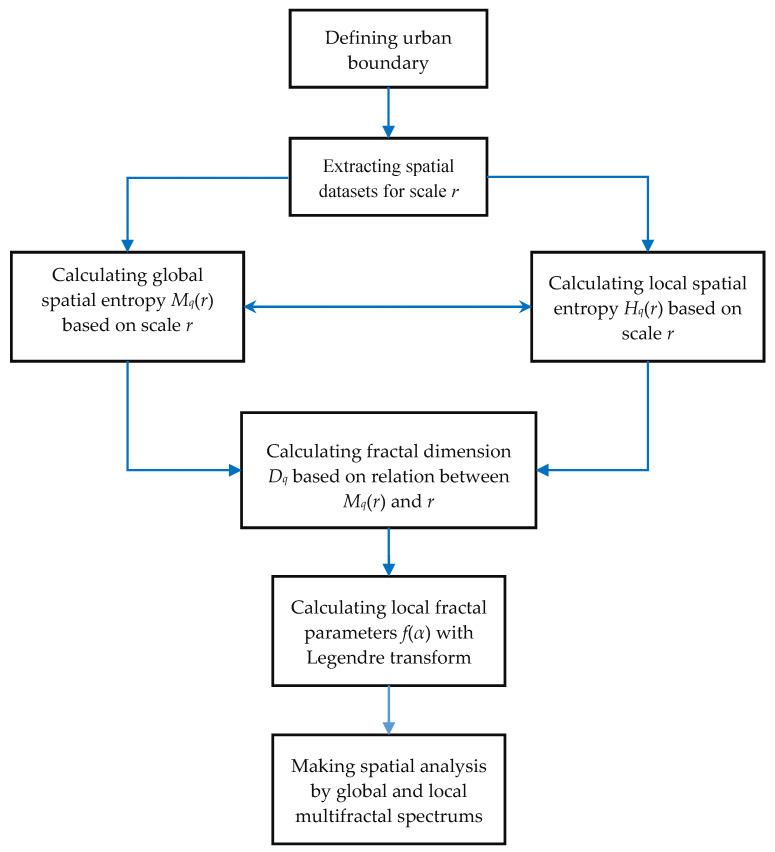
A flow chart of spatial analysis for cities from spatial entropy to multifractal spectrums. Note: Spatial entropy can be used to make spatial analysis of cities based on characteristic scales, while multifractal spectrums can be employed to make spatial analysis based on scaling in cities.

- **Step 1**: Defining an urban boundary based on the recent image. The most recent material we used was the remote sensing image of 2015. Based on this image, the boundary of Beijing city can be identified by using the “City Clustering Algorithm” (CCA) developed by Rozenfeld et al. [40,41]. The urban boundary can be called an urban envelope [15,32]. Then, a measure area can be determined in terms of the urban envelope [8].
- **Step 2**: Extracting the spatial datasets using the function box-counting method. First of all, we can extract the dataset from the image of the recent year (2015). A set of boxes is actually a grid of rectangular squares, each of which has an area of urban land use. The area may be represented by the pixel number. Therefore, in the dataset, each number represents a value of land-use area of the urban pattern falling into a box (square). Changing the linear size of the boxes, we will have different datasets. The box system forms a hierarchy of grids, which yield a hierarchy of spatial datasets. Applying the system of boxes to the images in different years, we have different datasets for calculating spatial entropy and fractal dimensions.
- **Step 3**: Calculate the spatial Renyi entropy and generalized Shannon entropy. Using Equations (5)–(8), we can compute the generalized entropies of urban land use based on a given linear size of functional boxes. For each linear size of boxes, we can obtain a Renyi entropy value or a generalized Shannon entropy value for Beijing’s urban form. For each year, we have a number of sets of entropy values based on different linear sizes of boxes. If the entropy values based on different box sizes have no significant differences, we can utilize the generalized entropy values to conduct a spatial analysis of urban form and growth.
- **Step 4**: Computing the multifractal parameter spectrums. If the entropy values depend heavily on the linear sizes of boxes, we should transform the Renyi entropy into the generalized correlation dimension using Equation (4). For different linear sizes of boxes *r*, we have different Renyi entropy values, which are defined as *M_q_*(*r*). As shown by Equation (5), there is a linear relation between ln(*r*) and *M_q_*(*r*). Similarly, we can convert the generalized Shannon entropy values into local multifractal dimension using Equations (7) and (8). By using Legendre transform, as shown in Equations (11) and (12), a complete set of multifractal parameters can be obtained, and multifractal spectrums can be generated. The computational and analytical process can be illustrated as follows (Figure 4).

The process of parameter estimation is simple by means of the least square calculations. Using a linear regression technique, we can estimate the generalized correlation dimension *D_q_*, which is just the slope of the semi-logarithmic equation. It should be noted that the regression equation has no intercept [34]. If *q* = 1, Equations (4) and (5) will be invalid. In this case, according to the L’Hospitale rule, the Renyi entropy will be replaced by the Shannon entropy, that is:

(13) $$H(r)=M_{1}(r)=-\sum_{i=1}^{N(r)}P_{i}(r)\ln P_{i}(r)$$

where *H*(*r*) denotes Shannon’s information entropy based on the linear size of boxes *r*. This implies that the Shannon entropy is the special case of the Renyi entropy, and the generalized Shannon entropy is shown above. Applying Shannon entropy to geographical analysis yields the important concept of spatial entropy [42]. In fact, Renyi’s entropy can be regarded as a kind of generalization of Shannon’s entropy. In short, for *q* = 1, Equation (5) will be substituted by the following relation:

(14) $$H(r)=-\sum_{i=1}^{N(r)}P_{i}(r)\ln P_{i}(r)=-D_{1}\ln r$$

which will give the information dimension of the multifractal dimension spectrums.

### 3.2. Results and Findings

**Figure 5:**
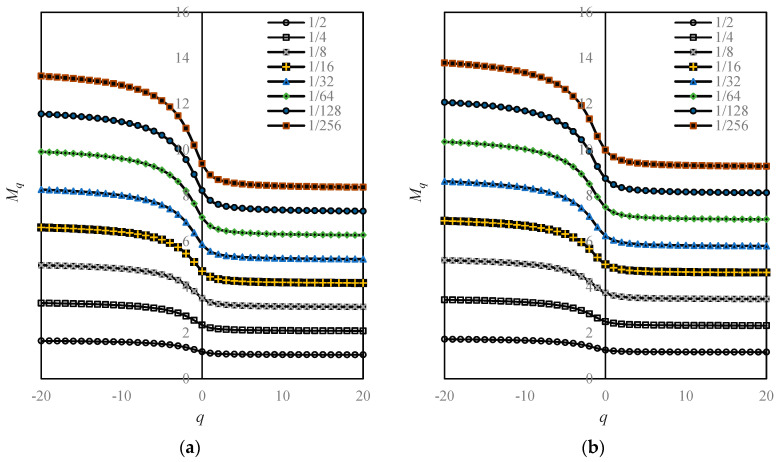

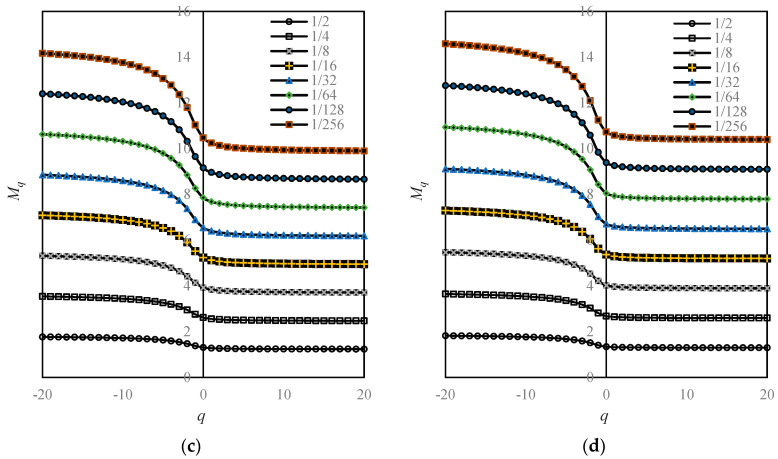
The Renyi entropy spectrums based on moment order parameter and different spatial scales of measurement. Note: From the bottom to the top, the linear size of functional boxes are *r* = 1/2, 1/4, 1/8, 1/16, 1/32, 1/64, 1/128, and 1/256, respectively. Different linear sizes of the boxes represent different spatial scales of measurements, and different Renyi entropy spectral lines based on different box sizes reflect the scale-dependence of spatial entropy. (**a**) 1984; (**b**) 1994; (**c**) 2006; and (**d**) 2015.

The above process of data extraction and parameter estimation is convenient by means of ArcGIS technique and mathematical computation software such as Matlab. The methods and steps have been illustrated in previous works [29,34]. Partial spatial Renyi entropy values for Beijing are shown in Table 3, and the corresponding multifractal parameters are displayed in Table 4. More results can be found in the attached files of Excel data (online supporting file). If the moment order *q* = 0, we have Hartley macrostate entropy; if *q* = 1, we have Shannon information entropy; if *q* = 2, we have Renyi correlation entropy. For an arbitrary order of moment *q*, we have Renyi’s generalized entropy. Obviously, for a given order of moment, say, *q* = 0, the entropy *M*_0_(*r*) value depends significantly on the linear sizes of boxes *r* (Figure 5, Table 3). In other words, the spatial Renyi entropy values of Beijing urban land use rely on the scales of measurement. Based on different linear sizes of boxes, the entropy values are different. In particular, the average value of the spatial entropy is invalid, because the mean depends on the size of datasets. That is to say, changing the range of the linear sizes of boxes yields different average values of Renyi entropy. Using Legendre transform, we can evaluate the corresponding generalized Shannon entropy and the mixed entropy.

**Figure 6:**
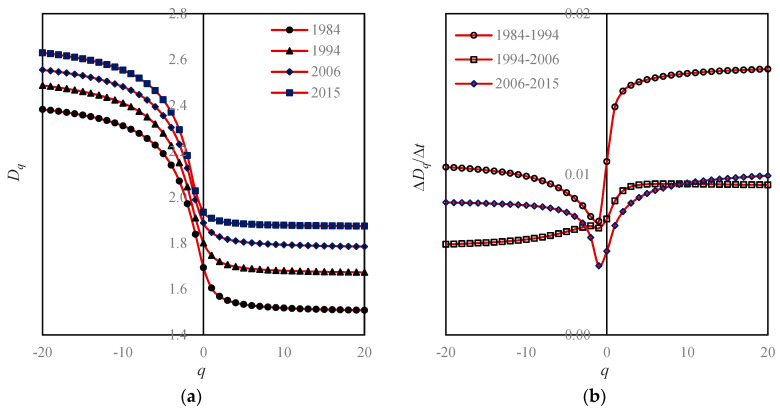
The global multifractal spectrums based on moment order parameter and change curves of global dimensions. Note: Based on different linear sizes of functional boxes, i.e., *r*=1/2, 1/4, 1/8, 1/16, 1/32, 1/64, 1/128, and 1/256, and the corresponding Renyi entropy *M_q_*(*r*) values, the generalized correlation dimension *D_q_* can be evaluated. Multifractal parameter values depend on moment order *q*, but are independent of spatial scale *r*. (**a**) Global multifractal dimension spectrums; (**b**) Global fractal dimension changes.

**Figure 7:**
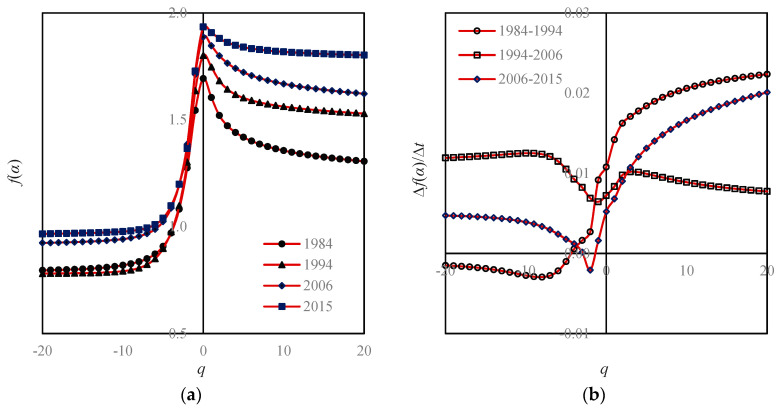
The local multifractal spectrums based on moment order parameter and change curves of local dimensions. Note: Based on different linear sizes of functional boxes, i.e., *r* = 1/2, 1/4, 1/8, 1/16, 1/32, 1/64, 1/128, and 1/256, and the corresponding generalized Shannon entropy *H_q_*(*r*) values, the generalized information dimension *f*(*α*) can be evaluated. (**a**) Local multifractal dimension curves; (**b**) Local fractal dimension changes.

**Figure 8:**
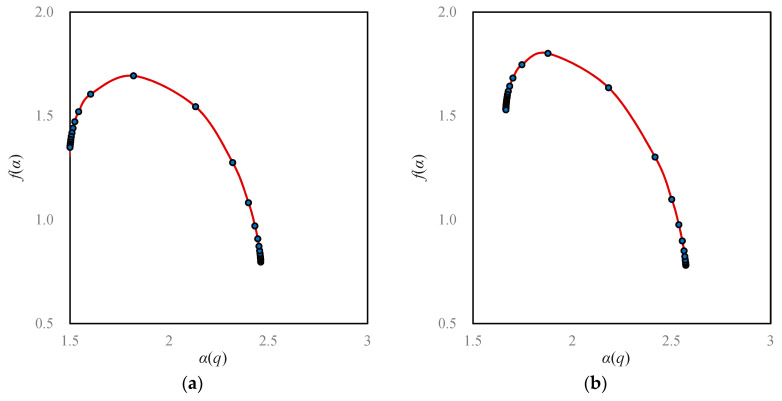

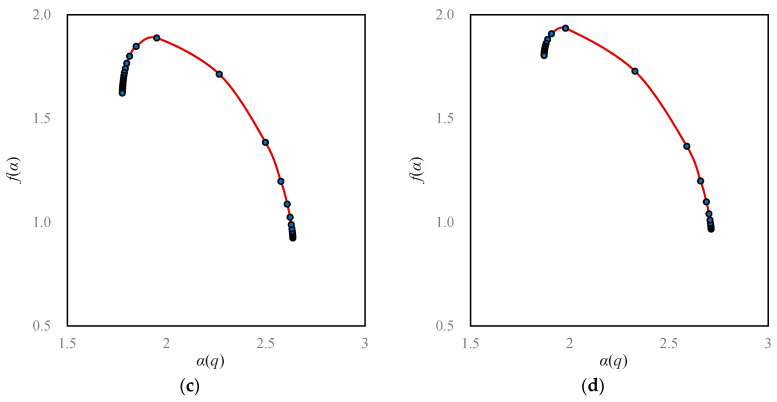
The local multifractal spectrums based on singularity exponent, i.e., the *f*(*α*) curves. Note: A local multifractal spectrum is a unimodal curve, which can be used to reflect the aggregation or diffusion of a city’s evolution. (**a**) 1984; (**b**) 1994; (**c**) 2006; (**d**) 2015.

If we convert the Renyi’s entropy values into multifractal parameters, the value of a parameter is unique. For the moment order *q* = 0, we can transform a series of Boltzmann macrostate entropy *M*_0_(*r*) values into a capacity dimension *D*_0_ value; For *q* = 1, we can transform a series of Shannon information entropy *M*_1_(*r*) values into an information dimension *D*_1_ value; for *q* = 2, we can transform a series of Renyi correlation entropy *M*_2_(*r*) values into a correlation dimension *D*_2_ value. For an arbitrary order of moment *q*, we can transform Renyi’s generalized entropy *M_q_*(*r*) values into a set of generalized correlation dimension *D_q_* values. Apparently, for a given order of moment, say, *q* = 1, the fractal dimension *D*_1_ value is independent of the linear sizes of boxes *r* (Figure 6). Using Equation (3), we can convert the generalized correlation dimension *D_q_* values into the mass exponent *τ_q_* values. As indicated above, the generalized correlation dimension *D_q_* and mass exponent *τ_q_* belong to the global parameters of multifractal models. By means of Legendre transform, Equations (11) and (12), we can transform the global parameters into local parameters, including the singularity exponent *α*(*q*) and the corresponding fractal dimension *f*(*α*(*q*)) (Table 4). Based on the global parameters, we have the global multifractal spectrum, i.e., *D_q_*-*q* spectrums (Figure 6); based on the local parameters, we have the local multifractal curves (Figure 7), and *f*(*α*)−*α* spectrums (Figure 8). The local spectrum is often termed an *f*(*α*) curve in the literature [18]. In practice, we can compute the local parameter values by using the normalized measure method first [26,27]. Then, using Legendre transform, we can convert the local parameter values into the global parameter values [29,34,43].

The main task of this article is not to explore the land-use patterns and processes of Beijing city. Instead, this paper is devoted to solving the problem regarding the scale dependence of spatial entropy and the related spatial heterogeneity description using fractal dimensions. Nevertheless, we still discuss the growth characteristics of Beijing by means of complexity measures. It is difficult to conduct a spatial analysis of the urban form of Beijing using spatial Renyi entropy and generalized Shannon entropy. Due to scale dependence of spatial measurements, the spectral curves of the Renyi entropy are dazzling (Figure 5). The case of the generalized Shannon entropy spectrums is similar to that of the Renyi entropy. In contrast, it is easy to make a spatial analysis using multifractal spectrums, because there is only one spectral line for a given fractal parameter in a given year. Thus, a family of Renyi entropy spectrum curves can be replaced by a global dimension spectrum curve, and a number of subplots can be replaced by a subplot (Figure 5 and Figure 6a). Similarly, a family of generalized Shannon entropy spectrums can be substituted with a local dimension spectrum (Figure 7a). The global multifractal parameters can be used to analyze the spatial correlation of urban evolution, while the local parameters can be employed to analyze the spatial heterogeneity of urban structure. Fractal dimensions can be utilized to measure the space filling extent, spatial uniformity, and spatial complexity [31,44]. It can be treated as a concise measure of land-use intensity.

According to the multifractal spectrums, the chief characteristics of Beijing’s urban form and growth are as follows.

- **First, Beijing’s space-filling speed was too fast, and space-filling extent was too high.** From 1984 to 1994 to 2006 and then to 2015, the capacity dimension *D*_0_ values increased from 1.6932 to 1.8011 and 1.8877 to 1.9346. By means of the formula *v* = *D*_0_/2, we can calculate the space-filling rate of urban form [31,44], *v*; the results were 0.8466, 0.9005, 0.9439, and 0.9673. In recent years, the level of space filling is close to the upper limit of one.
- **Second, spatial heterogeneity became weaker and weaker**. From 1984 to 1994 and 2006 to 2015, the information dimension *D*_1_ values went up from 1.6048 to 1.7467 and 1.8468 to 1.9081. Using the formula *u* = 1 − *D*_1_/2, we can calculate the spatial redundancy rate of urban form, *u*; the results were 0.1976, 0.1267, 0.0766, and 0.0459. The spatial redundancy rate is in fact an index of spatial heterogeneity. A reduction of redundancy indicates a weakening process of spatial heterogeneity.
- **Third, the urban growth of Beijing is characterized by stages.** In the mass, the space-filling speed in the central area was obviously faster than that of the edge area (Figure 6 and Figure 7). Where the global feature is concerned, the characteristics are as below: From 1984 to 1994, the land-use speed in the central urban area was significantly higher than that in the fringe area; From 1994 to 2006, the gap of land-use speed between the central and peripheral areas decreased; from 2006 to 2015, the speed of land use in the central and peripheral areas was close to equilibrium (Figure 6b). Where the local level is concerned, the features are as below: From 1984 to 1994, the land-use speed in high-density areas was significantly higher than that in low-density areas. From 1994 to 2006, the situation reversed, and the land-use speed in low-density areas was significantly higher than that in high-density areas. From 2006 to 2015, the land-use speed in high-density areas was once again higher than that in low-density areas (Figure 7b).
- **Fourth, the growth of Beijing city is of outward expansion**. On the whole, the closer to the center area, the faster the space-filling speed will be. In terms of local fractal spectrums, city development can be classified into two types: one is central aggregation, and the other is peripheral expansion [43]. The difference can be reflected by the local multifractal spectrums. The unbalance of urban spatial expansion leads to the asymmetry of *f*(*α*) curves. If the urban development is centralized, the peak of the spectral curve tilts to the right; on the contrary, if the urban development is characterized by periphery diffusion, the peak of the spectrum inclines to the left [43]. The peak values of Beijing’s *f*(*α*) curves are obviously left-sided, which imply that Beijing’s development is mainly a process of expanding to the periphery (Figure 8).
- **Fifth, there was redundant correlation in Beijing’s urban fringe**. Generally speaking, the generalized correlation dimension value lies between zero and two. However, when the order of moment *q* approaches negative infinity, the *D_q_* values exceeded two, and became bigger and bigger (Figure 6). This suggests that there are too many messy patches of land use to fill the urban fringe.
- **Sixth, the quality of spatial structure of Beijing city declined**. A local multifractal spectrum is supposed to be a smooth single-peak curve. In 1984, the local fractal dimension spectral lines were regular. However, from 1995 to 2015, the *f*(*α*) curves deviated more and more from the normative spectral line (Figure 8).

## 4. Discussion

**Figure 9:**
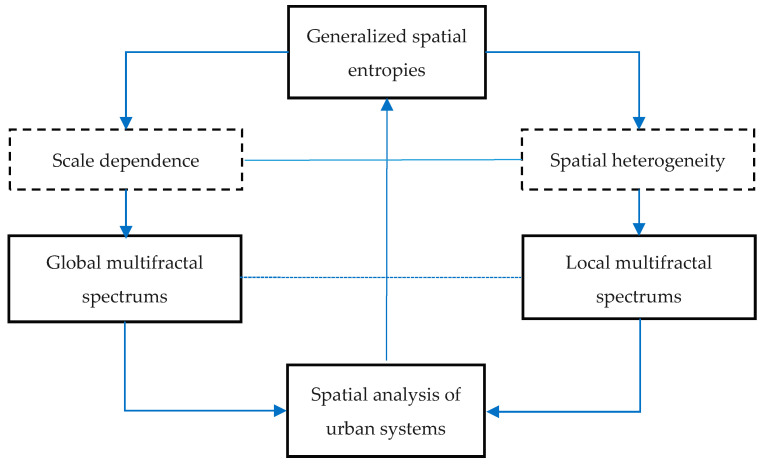
Two cases of spatial entropy analyses transformed into fractal dimension analyses. Note: The problem of scale dependence of entropy measurement can be solved by transforming the generalized entropies into fractal dimensions by means of scaling relation, and the property of spatial heterogeneity can be characterized by multifractal parameter spectrums.

Entropy and fractal dimension are two important measures of spatial complexity in the geographical world. Substituting generalized spatial entropy by multifractal parameters, we can solve two problems for urban studies. One is the scale dependence of entropy measurements, and the other is the description of the spatial heterogeneity of urban morphology. In particular, if we convert spatial entropy into a fractal dimension, a number of entropy values based on different scales can be represented by one fractal dimension, which is independent of scales. Thus, many numbers are condensed into one number, so that the description and analytical process will become simpler. These properties have been illustrated by the above case study of Beijing city. In fact, using the scaling relations between the linear scales of measurement and the measure results, we can transform various entropies and entropy spectrums into fractal parameters and multifractal dimension spectrums. The global multifractal parameters and the corresponding entropies can be related to the local multifractal parameters and the corresponding entropies by Legendre transform (Table 5). As a result, the fractal models can associate spatial correlation functions with entropy functions [43]. Therefore, based on fractal dimensions, the concept of scale dependence is replaced by the notion of spatial dependence. Spatial dependence (spatial correlation) and spatial heterogeneity (spatial difference) reflect two essential aspects of geographical systems [45,46]. For a simple system, the spatial entropy has a determinate value. However, for a complex system, such as system of cities, the values of spatial entropy depend on the scales of measurement; thus, we cannot find a certain entropy value for urban form and urban systems. It is advisable to transform spatial entropy values into the corresponding fractal parameters. On the other hand, multifractal scaling provides a quantitative characterization of heterogeneous phenomena [10]. If we want to explore spatial heterogeneity deeply in a complex spatial system such as system of cities, the limitation of entropy will also appear. Due to entropy conservation, different parts of a fractal urban system bear the same entropy value. So, we cannot bring to light the local features by spatial entropy. In this instance, we can use multifractal parameters to characterize the spatial heterogeneity of urban form and urban systems (Figure 9).

The measurement of spatial entropy has a natural connection with the fractal dimensions of urban systems. In the literature, both entropy and fractal dimensions have been employed to characterize urban patterns and evolution process [47,48,49,50]. However, the scale dependence of generalized spatial entropies and their relationships with multifractal dimension spectrums are rarely reported. The scale dependence of spatial entropy measurements is associated with the scale-free property of urban systems. Fractal dimension can be used to act as the characteristic parameter of urban description. This problem has been preliminarily researched in previous works [8,25]. In one companion paper, using the box-counting method, we revealed that spatial entropies depend on the scales of measurement, and the normalized entropy values are empirically equal to the normalized fractal dimension values [8]. This suggests that two approaches can be utilized to solve the problem of the scale dependence of spatial entropy. One is to use fractal dimensions to replace spatial entropies, and the other is to normalize spatial entropies. Three typical fractal dimensions in global multifractal dimensions, i.e., capacity dimension *D*_0_, information dimension *D*_1_, and correlation dimension *D*_2_, are discussed in this research, but the results have not been generalized to multifractal parameter spectrums. In another companion paper, based on area radius scaling, the normalized Renyi entropy is generalized to multifractal spectrums [25]. Two sets of multifractal indicators are proposed to describe urban growth and form. The mathematical modeling based on characteristic scales and the spatial analysis based on scaling are integrated into a logical framework. Compared with the previous studies, this work bears three new points. First, the scale dependence of spatial Renyi entropy and generalized Shannon entropy is illustrated by the box-counting method. Changing the linear sizes of boxes yields different entropy spectral curves for the Renyi entropy and generalized Shannon entropy. It is complicated to conduct a spatial analysis of cities using these curves of entropy spectrums. Second, the solution to the scale-dependence problem of spatial entropies is clarified. Transforming the Renyi entropy into global multifractal parameters and converting the generalized Shannon entropy into local multifractal parameters, the different entropy values based on different measurement scales will be replaced by two fractal dimension values, which are actually characteristic values of generalized spatial entropies and independent of scales of measurement. Third, similarities and differences between spatial entropy and fractal dimension spectrums are illustrated. Spatial entropy is simple and easy to understand, but it cannot be used to describe the spatial heterogeneity of city systems. In contrast, using multifractal parameter spectrums, we can characterize the spatial heterogeneity of urban forms and urban systems. Unfortunately, multifractal spectrums are not suitable for non-fractal systems. The main shortcomings of this work rest with two aspects. First, the empirical analysis is chiefly based on the box-counting method. The other methods, such as the sandbox method, growing cluster method, and so on, are not taken into account for the time being. All of these methods can be applied to the studies on fractal cities. Second, the research method is confined to the fractal cities that are defined in the two-dimensional embedding space. If we take the third dimension of urban space, the measurements and subsequent calculations are significantly limited. The solution to this problem is to develop a three-dimensional box-counting method of fractal dimension estimation. What is more, the uncertainty of fractal dimension is not discussed. The fractal dimension values of urban form and urban systems depend on the size and central location of a study area.

## 5. Conclusions

Fractal dimensions are based on entropy functions, and this suggests that the generalized spatial entropies can be associated with the fractal dimensions of cities. According to the theoretical exploration and empirical analysis, the main conclusions of this paper can be reached as follows. First, multifractal dimensions can be used to solve the problem of the scale dependence of the generalized spatial entropies of cities. For the simple spatial systems, we can obtain determinate entropy values. However, for complex spatial systems such as cities and systems of cities, we cannot gain certain entropy values. Both the generalized Shannon’s information entropy and the Renyi entropy spectrums depend on the scales of measurement. The uncertainty of entropy values give rise to trouble for spatial modeling and the quantitative analysis of cities. One effective method of solving the problem is to substitute the spatial entropies with fractal parameters. Fractal dimension values do not depend on the scales of measurement. We can use the capacity dimension to replace Hartley’s macrostate entropy, the information dimension to replace Shannon’s entropy, the generalized correlation dimension spectrum to replace Renyi’s entropy spectrum, and the local multifractal spectrums to replace the generalized Shannon entropy spectrums. Second, multifractal scaling can be employed to describe the spatial heterogeneity of cities. The scale dependence indicates fractals and scaling. Simple fractal systems have homogeneous structures, in which different parts have the same entropy and fractal dimensions. However, complex spatial systems such as cities and systems of cities have heterogeneous structures, in which different parts have different local fractal dimension values, but have the same entropy value. This suggests that generalized entropy values cannot reflect the spatial differences of complex spatial systems such as cities. In contrast, multifractal dimension spectrums can be used to reveal the spatial heterogeneity of complex systems, including urban form and urban systems. A global dimension spectrum can better reflect spatial dependence, while a local dimension spectrum can more effectively describe spatial differences. Among various multifractal parameters, the spatial redundancy rate based on the information dimension can be used as a concise index of the spatial heterogeneity of cities.

## Figures and Tables

**Table 1:** The values of entropy and fractal dimension of a regular growing monofractal system.

Step for Fractal Generation	Figure 1a: Variable Size of Study Area and Measurement Scale		Figure 1b: Fixed Size of Study Area and Variable Measurement Scale	
	Entropy (nat)	Fractal Dimension	Entropy (nat)	Fractal Dimension
1 (outlier)	0	0	0	2
2	1.6094	1.4650	1.6094	1.4650
3	3.2189	1.4650	3.2189	1.4650
4	4.8283	1.4650	4.8283	1.4650
…	…	…	…	…
*m*	ln(5*^m^*^−1^)	ln(5*^m^*^−1^)/ln(3*^m^*^−1^)	ln(5*^m^*^−1^)	ln(5*^m^*^−1^)/ln(3*^m^*^−1^)

**Note**: Different steps reflect different levels in a fractal hierarchy.

**Table 2:** The values of entropy and fractal dimension of a regular growing multifractal system.

Step for Fractal Generation	Global Feature		Local Features			
			Central Part		Peripheral Parts	
	Entropy (nat)	Fractal Dimension	Entropy (nat)	Fractal Dimension	Entropy (nat)	Fractal Dimension
**1 (outlier)**	0	0	0	-	0	-
**2**	1.5285	1.7604	0.1667	1.7604	1.3618	1.5791
**3**	3.0569	1.7604	1.5285	1.7604	1.5285	1.5791

**Table 3:** Partial generalized entropy values of Beijing’s urban land-use pattern in 2015.

Moment Order *q*	Generalized Entropy Based on Different Scales *r*							
	*r* = 1/2	*r* = 1/4	*r* = 1/8	*r* = 1/16	*r* = 1/32	*r* = 1/64	*r* = 1/128	*r* = 1/256
−20	1.8225	3.6449	5.4674	7.2898	9.1123	10.9347	12.7572	14.5797
−15	1.8045	3.6089	5.4134	7.2179	9.0223	10.8268	12.6313	14.4357
−10	1.7702	3.5405	5.3107	7.0809	8.8511	10.6214	12.3916	14.1618
−5	1.6806	3.3612	5.0417	6.7223	8.4029	10.0835	11.7640	13.4446
−4	1.6430	3.2859	4.9289	6.5718	8.2148	9.8578	11.5007	13.1437
−3	1.5901	3.1801	4.7702	6.3602	7.9503	9.5403	11.1304	12.7204
−2	1.5123	3.0246	4.5369	6.0493	7.5616	9.0739	10.5862	12.0985
−1	1.4058	2.8116	4.2174	5.6232	7.0290	8.4348	9.8406	11.2464
0	1.3410	2.6820	4.0230	5.3640	6.7050	8.0460	9.3870	10.7280
1	1.3226	2.6452	3.9679	5.2905	6.6131	7.9357	9.2583	10.5809
2	1.3148	2.6296	3.9443	5.2591	6.5739	7.8887	9.2035	10.5183
3	1.3105	2.6209	3.9314	5.2419	6.5523	7.8628	9.1732	10.4837
4	1.3078	2.6155	3.9233	5.2311	6.5388	7.8466	9.1544	10.4621
5	1.3059	2.6119	3.9178	5.2237	6.5297	7.8356	9.1416	10.4475
10	1.3017	2.6035	3.9052	5.2069	6.5087	7.8104	9.1122	10.4139
15	1.3001	2.6003	3.9004	5.2006	6.5007	7.8008	9.1010	10.4011
20	1.2993	2.5986	3.8979	5.1972	6.4965	7.7957	9.0950	10.3943

**Note**: For *q* = 1, the numbers represent Shannon’s information entropy values.

**Table 4:** Partial multifractal parameter values of Beijing’s urban land-use pattern in 2015.

Moment Order *q*	Fractal Parameter and Goodness of Fit						
	*D_q_*	*R* ^2^	*τ_q_*	*α*(*q*)	*R* ^2^	*f*(*α*)	*R* ^2^
−20	2.6293	0.8308	−55.2143	2.7124	0.8113	0.9664	0.7092
−15	2.6033	0.8369	−41.6527	2.7122	0.8115	0.9698	0.7112
−10	2.5539	0.8484	−28.0929	2.7116	0.8120	0.9771	0.7159
−5	2.4246	0.8790	−14.5473	2.7015	0.8116	1.0397	0.7361
−4	2.3703	0.8927	−11.8515	2.6884	0.8082	1.0979	0.7423
−3	2.2940	0.9137	−9.1759	2.6592	0.8012	1.1983	0.7535
−2	2.1818	0.9470	−6.5454	2.5898	0.7984	1.3658	0.8074
−1	2.0281	0.9874	−4.0563	2.3288	0.8782	1.7275	0.9683
0	1.9346	0.9992	−1.9346	1.9791	0.9951	1.9346	0.9992
1	1.9081	1.0000	0.0000	1.9081	1.0000	1.9081	1.0000
2	1.8968	0.9998	1.8968	1.8888	0.9994	1.8807	0.9987
3	1.8906	0.9995	3.7812	1.8810	0.9986	1.8618	0.9959
4	1.8867	0.9992	5.6601	1.8773	0.9982	1.8489	0.9927
5	1.8841	0.9989	7.5363	1.8752	0.9979	1.8399	0.9900
10	1.8780	0.9982	16.9021	1.8720	0.9974	1.8181	0.9824
15	1.8757	0.9979	26.2599	1.8712	0.9973	1.8086	0.9784
20	1.8745	0.9978	35.6151	1.8709	0.9972	1.8031	0.9757

**Note**: The global parameter values are estimated using Equations (6)–(8), while the local parameters are estimated by means of the *μ*-weight method.

**Table 5:** The correspondence relationships between entropies and fractal dimensions for the spatial analysis of cities.

**Parameter Level**		**Entropy**	**Fractal Dimension**
Global parameter	General	Renyi entropy spectrum	Global multifractal dimension spectrum
	Special	Hartley entropy	Capacity dimension
		Shannon entropy	Information dimension
		The second-order Renyi entropy	Correlation dimension
**Connection**		**Legendre Transform**	
Local parameter	General	Generalized Shannon entropy spectrum	Local multifractal dimension spectrum
		Mixed entropy	Singularity exponent spectrum
	Special	Hartley entropy	Capacity dimension
		Shannon entropy	Information dimension

**Note**: The Hartley entropy and Shannon entropy represent the intersection of the Renyi entropy and the generalized Shannon entropy; thus, the capacity dimension and information dimension form the intersection of the global multifractal dimensions and local multifractal dimensions.
